# Optimization of the Speech Test Material in a Group of Hearing Impaired Subjects: A Feasibility Study for Multilingual Digit Triplet Test Development

**DOI:** 10.3390/audiolres11030032

**Published:** 2021-07-12

**Authors:** Marcin Masalski, Martyna Adamczyk, Krzysztof Morawski

**Affiliations:** 1 Department of Otolaryngology Head and Neck Surgery, Wroclaw Medical University, 50-367 Wroclaw, Poland; mkruszynska@usk.wroc.pl; 2 Institute of Biomedical Engineering and Instrumentation, Wroclaw University of Technology, 50-370 Wroclaw, Poland; 3 Department of Otolaryngology, Institute of Medical Sciences, University of Opole, 45-401 Opole, Poland; morawski@neurotology.pl

**Keywords:** speech audiometry, speech intelligibility in noise, digit triplet test, digit-in-noise test, speech reception threshold, tele-audiology, mobile-based hearing tests

## Abstract

Background: The development of the global digit-in-noise test requires optimization of each language version on a group of normal-hearing native-speakers. An alternative solution may be an adaptive optimization during ongoing tests in a group of subjects with unknown hearing impairments. The objective of the research was to compare the optimization results between these groups. Methods: Digit triplets consisting of three pseudo-randomly selected digits were presented in speech-shaped noise at various signal-to-noise ratios (SNRs), according to the protocol of the final speech test. Digit-specific and position-specific speech reception thresholds (SRTs) were determined and compared between groups. Results: The study sample consisted of 82 subjects, 26 normal-hearing subjects and 56 patients with diverse hearing disorders. Statistically significant differences in digit-specific SRTs between the control and the investigated group were obtained for three digits in continuous noise (digits 0, 4, 6; *p*-value of 0.04, 0.03, 0.05) and two in modulated noise (digits 1 and 6; *p*-value of 0.05 and 0.01). An analysis including only ears with SRTs within the range of the normal hearing control group showed no statistically significant differences between digits. Conclusion: Optimization of speech material can be carried out in a group of subjects with unknown hearing impairments, provided the ears with scores outside normal range are rejected.

## 1. Introduction

The burden of hearing loss is common worldwide. Communication difficulties, low self-esteem, and isolation lead to social exclusion and depression [1,2,3]. Currently, worldwide hearing loss prevalence is estimated between 4.0% and 18.1% [1,4,5,6,7,8]. Despite significant variation in the estimated values resulting primarily from the adopted methodology, the prevalence increases globally due to the aging of the worldwide population and the strong positive correlation between hearing impairment and age [4,5,9]. Therefore, extensive measures are taken in the field of prevention, diagnosis and treatment of hearing loss [5].

Mobile-based hearing tests can be carried out at low cost and on a large scale [10,11,12,13,14,15,16,17,18]. They are applied as screening tests, especially in underserved areas, in preliminary evaluation of audiological patients, in self-monitoring of hearing or even in epidemiological studies [8,16,17,19,20,21,22]. Such applications increase awareness of hearing loss and motivate to take individual and systemic preventive measures [5,23].

Mobile-based hearing tests implemented by means of tonal signals can be easily adopted for global usage and some of them are already available worldwide [10,12,14,15,18], unlike mobile-based speech tests that are available for selected languages only [24,25,26]. Comparison of test results between various languages, as opposed to tonal-based tests, is much more complex due to the differences in speech material, presentation methods, test algorithms and scoring procedures.

Guidelines for the development of internationally comparable screening tests for assessing speech intelligibility are presented in [23,27,28,29]. The starting point was the digit triplet test developed in Dutch [30] for telephone use and then in the Internet version [31]. The test consists in identifying three digits presented in noise at different signal-to-noise ratios. The advantage of digit triplets is the use of words commonly known in the population, the inability to deduce responses from the context and learn by heart, as well as the minimal impact of the cognitive abilities on the results [30,32]. Digit triplets also reveal very high correlation with the sentence test, despite not reflecting the general phoneme distribution of a given language [30,33]. In addition to the Dutch language [30,31], the versions of the triplet test were also prepared for Canadian English [34,35], Danish [36], American English [37,38], German [33,39,40,41], British English [42,43], Polish [32], French [44], New Zealand English [45], Swedish and Greek (unpublished, given after [23]), Finnish [46], Russian [47], Malay [48], South African English [24], Canadian French [35], Persian [49] and Korean [50]. The multiplicity of language versions indicates digit triplet test usability. However, the above constitutes only a small percentage of the world’s languages and reflects the need for improvement of test development procedures.

Speech audiometry in noise measures speech reception threshold (SRT) that determines a signal-to-noise ratio (SNR) at which a given ratio (commonly 50%) of the presented items (sentences, words or digits) are recognized correctly. The smaller the standard deviation of the SRT in a given item sample, the more efficient the test [51,52]. The optimization of the test consists in minimizing the SRT deviation by determining correction coefficients of item-specific SRTs so that the intelligibility of each item is similar [23]. The optimization is carried out in a group of normal-hearing native-speakers due to relatively small inter-individual SRT. Nevertheless, the measurements are tedious and hours-long and consequently difficult to organize for many languages. There is an alternative to normal-hearing native-speaker optimization, as it may be substituted by an adaptive procedure that is applied during ongoing testing using the results of subjects with an unknown hearing threshold, thus improving the efficiency of the test as new results become available. This semi-automatic optimization of language versions can be easily adopted into already globally available solutions for hearing testing. However, the adaptive optimization is feasible only if the item-specific SRT differences in normal-hearing subjects are consistent with those in hearing-impaired subjects. As the literature data on these differences are limited and ambiguous [38,49,53,54] more detailed studies are required. In the case of the digit triplet test, besides the digit-specific SRT, the digit position-specific SRT must also be considered.

The efficiency of speech audiometry tests, apart from the SRT variability, also depends on the slope of the psychometric function that determines the intelligibility ratio in relation to SNR. The steeper the slope, the more efficient the test [23,55]. The slope, in contrast to the SRT, is not subject to simple adjustment as it is a distinctive feature of the speech material itself. The improvement of the test efficiency can, however, be achieved by rejecting the words with the shallowest slopes [28,30,32,46,56]. In the optimization of multilingual digit triplet test, it will be feasible to reject a digit based on the slopes measured in a group of subjects with unknown hearing loss only if the values are consistent with those of normal-hearing subjects.

The aim of the study was to assess the optimization feasibility of the digit triplet test in a group of subjects with diverse hearing disorders. The evaluation was carried out by comparing digit-specific and position-specific SRTs, as well as digit-specific slopes between a normal-hearing subject group and the group of subjects with diverse hearing disorders.

## 2. Materials and Methods

The study was a single-center, parallel, closed trial carried out on employees, students and patients of the ENT Department. The consent to conduct the trial has been given by the Bioethics Committee of Wroclaw Medical University. The subjects were recruited offline using face-to-face prompting. Prior to the study, the participants were informed of the purpose and course of the study, and they had signed the informed consent.

### 2.1. Speech Material

Speech material composed of 10 Polish digits spoken by a male native-speaker lector. All monosyllabic (2,3,5,6) and disyllabic (0,1,4,7–10) digits were used. The material was recorded in a recording studio using Neumann U87 condenser microphone and Audient ASP008 preamplifier, and then subjected to digital conversion through an analogue-digital converter MOTU 2408mk3. The conversion was carried out at a sampling frequency of 44.1 kHz and with 24-bit resolution. Fragments of the recorded material (about 1 s in length) containing digits preceded by 50 ms of silence were extracted and saved in separate files.

The masking noise was created by digital filtering of a white noise. A FIR filter of the order of *n* = 500 was used. The filter was designed to reflect the frequency of the signal formed from the combination of all digits. The resulting speech-shaped noise of 5 s in length [30,42,43] was saved to a file.

As the test was intended to be used on personal computers and mobile devices, the sound files were subjected to lossy compression to the MPEG-1 Audio Layer II format with a sampling frequency of 44.1 kHz and 16-bit resolution. The compression significantly reduces the file size while only slightly lowering the quality of the recording. Lower quality may decrease the intelligibility of the speech material, but it can also be an advantage in standardizing the test when presented on various-quality equipment.

The triplets were generated and played back by an applet implemented in Java running in a browser. Each triplet was generated on the fly during testing using ten digit sound files and one speech-shaped-noise sound file. The sound files were loaded from a server just after the applet was started. Each triplet consisted of 3 digits generated pseudo-randomly without repetitions and presented in noise: continuous or modulated. The continuous noise was chosen from a cyclic buffer of 5 s in length containing speech-shaped noise at a pseudo-random position. The modulated noise was created from continuous noise using rectangular envelope of 20 dB depth and modulation frequency of 16 Hz [52]. A constant shift of the noise modulation relative to the digit was provided. The noise was presented at a fixed intensity level, while the amplitude of a digit was determined each time to obtain the desired SNR. The root mean square (RMS) for the digit was calculated between the first and last signal sample, whose amplitude exceeded 3% of the maximum value. Each digit was preceded by a 0.5 s noise fragment and followed by a 0.25 s fragment. A rising slope of 0.1 s at the beginning of the noise of the triplet and a descending slope of 0.1 s at its end were applied.

### 2.2. Measurement

The hearing threshold of each subject was determined by means of pure-tone audiometry. Pure-tone audiometry was performed using conventional 10 dB down and 5 dB up bracketing method in accordance with the standards of the British Society of Audiology [57]. The measurements were carried out by an audiologist using an Interacoustic AD229e clinical audiometer with TDH-39 and B71 headphones previously calibrated in accordance with ISO 389-1: 1998. The control group was recruited among employees and students and included ears with an air conduction (AC) hearing threshold lower or equal to 20 dB HL in the range between 250 Hz and 8 kHz [38]. The investigated group consisted of patients of the ENT Department, regardless of the hearing threshold.

Each subject performed the digit triplet test in continuous and modulated noise. The evaluation was carried out in the range from −26 to −6 dB SNR and from −32 to −12 dB SNR for continuous and modulated noise, respectively. The SNR ranges were determined on the basis of preliminary measurements to obtain extreme intelligibility values at their ends. Triplets were presented starting from the highest signal-to-noise ratio in 2 dB steps. The study participants were instructed to identify each digit of a triplet by pressing one of the buttons 0–9 or pressing the X button when the digit was completely incomprehensible. The entire triplet was then confirmed using the OK button. Digit scoring method was used. Participants without computer literacy were assisted by a technician. The measurement was conducted separately for each ear, starting with the right ear. Testing at the specified SNR was completed on one of three conditions: after 7 triplets (21 digits) were evaluated, when all the digits in the first three triplets were identified correctly, or when all the three digits in the first three triplets were identified incorrectly. The measurement was terminated after reaching a SNR level for which all the digits in the first three triplets were incomprehensible. No training was conducted prior to the test.

The digit triplet test was carried out in a sound booth of the ENT Department auditory lab, on a Lenovo T420s laptop equipped with common headphones (Media-Tech). The sound intensity was determined at the beginning of each examination, individually for each participant, at most comfortable listening level and could be changed during the test.

### 2.3. Statistical Analysis

The SRT was calculated for each digit and for each position of the digit in the triplet by fitting a psychometric function (Equation (1)) to the measurement data by means of least squares [32,54].

(1) $$\phi (SNR)=p_{0}+(1-p_{0})\cdot \mathrm{CDF}(SNR-SRT)$$

where: ϕ—psychometric function, SNR—signal-to-noise ratio, $p_{0}$—probability of random selection, SRT—speech reception threshold, CDF—normal cumulative distribution.

Digit-specific SRTs and position-specific SRTs determined in the control group were compared with the investigated group. Group differences were evaluated by means of confidence intervals for two independent samples after Bonferroni correction for multiple comparison. Bonferroni correction was applied to account for simultaneous comparison of 3 positions and simultaneous comparison of 10 digits. Confidence intervals were calculated by means of bootstrapping assuming the number of replicates at the level of 1000. The analyses were carried out using MATLAB R2018a (The MathWorks, Inc., Natick, MA, USA).

The sample size was estimated based on preliminary measurements of the digit-specific SRT resulting in a standard deviation of 1.9 dB and 2.6 dB in the control group and the investigated group, respectively. For a statistical significance level of 0.05, the statistical power of 0.8 and the effect size of 0.75 dB, the sample size was 50 and 95 ears in the control group and the investigated group, respectively. The research was completed after obtaining the required sample size in both groups.

### 2.4. Adjusted and Additional Analysis

Digit-specific SRTs and position-specific SRTs determined in the control group were also compared against the subgroup of investigated group having SRTs within normal range. The upper limit of the normal range was determined as the 99th percentile of the control group. Group differences were evaluated as before, using bootstrapping-calculated confidence intervals compared after applying Bonferroni correction.

An additional analysis, analogous to the SRT comparison, was also conducted for the slope of the psychometric function. The slope was determined for the digit and its position in the triplet on the basis of a derivative of the fitted psychometric function (Equation (2)). The value of the derivative was calculated algebraically at the SRT, directly from the definition of the derivative.

(2) $$S=\frac{d\phi (SRT)}{dSNR}$$

where: S—slope of psychometric function, ϕ—psychometric function, SNR—signal-to-noise ratio, SRT—speech reception threshold.

## 3. Results

**Figure 1:**
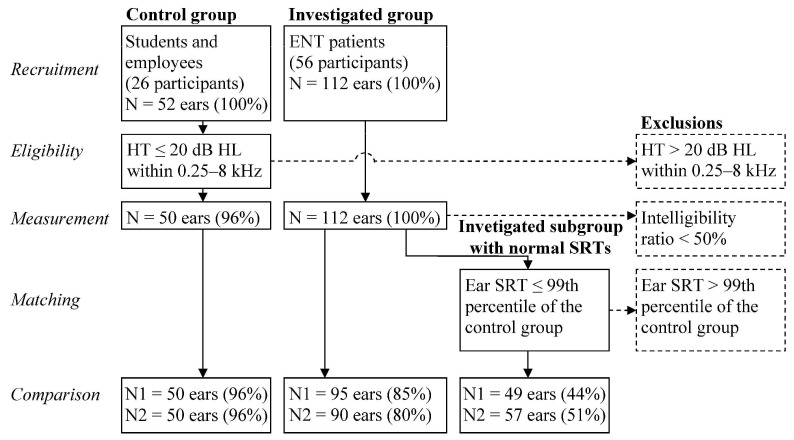
Attrition diagram (HT—air conduction hearing threshold, SRT—speech reception threshold, N1, N2—number of ears for continuous and modulated noise, respectively).

In the period from 13 June 2019 to 23 January 2020, 82 subjects (164 ears) were examined of which 26 were students and employees aged 18–36 (median 26.5) and 56 were patients aged 21–71 (median 40.0). The control group included 50 out of 52 ears (96%) from the group of students and employees for whom the AC hearing threshold in the entire frequency range from 250 to 8 kHz did not exceed 20dB HL. All the patients (112 ears) were included in the investigated group (Table 1, Figure 1).

**Figure 2:**
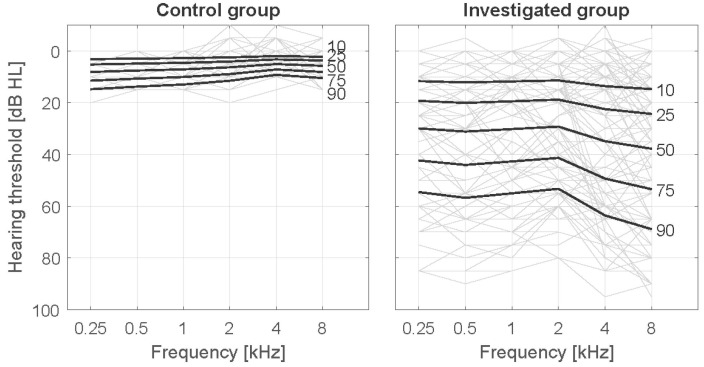
Air conduction hearing thresholds in the control group and in the investigated group.

The average AC hearing threshold at 0.25–8 kHz was 6.3 and 30.0 dB HL in the control group and in the investigated group, respectively. AC hearing thresholds are shown in Figure 2 and types of hearing loss in Table 2.

### 3.1. Speech Reception Threshold

**Figure 3:**
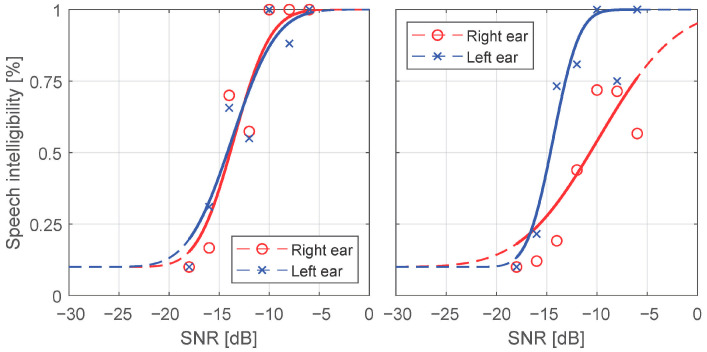
Examples of the speech intelligibility ratio in relation to the signal-to-noise ratio (SNR) fitted with the psychometric function for continuous noise (right panel—a subject of the control group, left panel—a subject of the investigated group with right-sided hearing impairment).

Speech intelligibility was calculated by dividing the number of correctly identified digits by the number of all the digits presented at a particular SNR. If the digit was incomprehensible and the X button was pressed, the numerator was increased by the probability of random selection at 1/10. Examples of speech intelligibility ratio in relation to SNR are shown in Figure 3. SRTs were calculated for all measurements for which speech intelligibility ratio exceeded 50%, i.e., for all ears in the control group and for 95 of 112 (84.8%) and 90 of 112 (80.4%) ears in the investigated group for continuous and modulated noise, respectively. Measurements for which the maximum speech intelligibility ratio was lower than 50% were rejected due to a substantial error of extrapolation. The results were checked for the SRT differences between the right and the left ear. No statistically significant differences were obtained (*p* = 0.94, d = 0.04 dB and *p* = 0.78, d = 0.17 dB for continuous and modulated noise, respectively).

**Figure 4:**
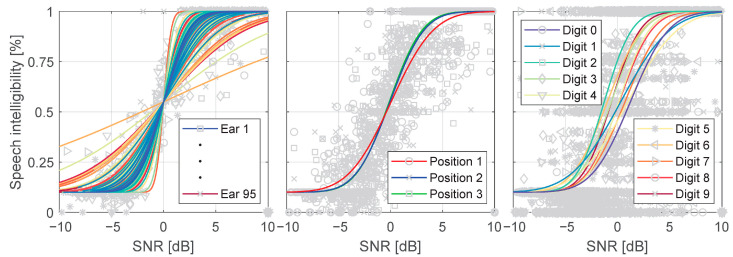
Example of psychometric functions for modulated noise in the investigated group: psychometric functions adjusted for ear SRT (left panel), position-specific (middle panel) and digit-specific (right panel) psychometric functions.

Speech intelligibility data were adjusted for inter-individual differences, separately for each ear basing on the SRT value of that ear. After adjustment, psychometric functions were determined in each group for the digits and their positions in the triplet (Figure 4). Digit-specific and position-specific SRTs were calculated using the psychometric function for continuous and modulated noise (Appendix A).

**Figure 5:**
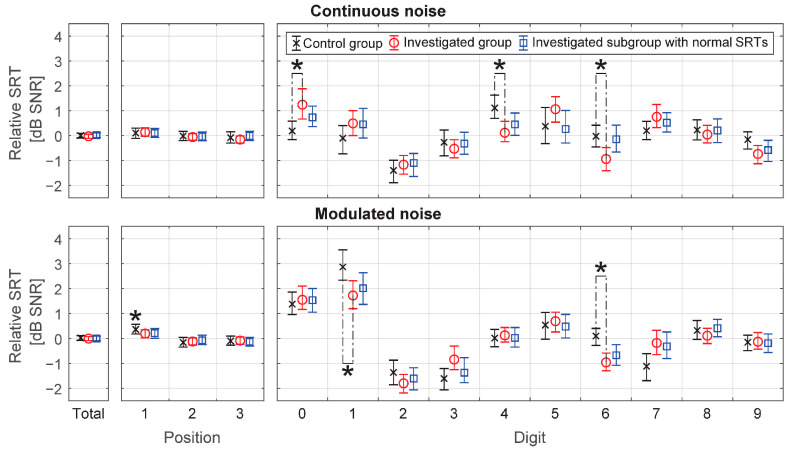
Position-specific and digit-specific speech reception thresholds (SRTs) for continuous and modulated noise. The Y-axis represents SRT normalized for individual differences (after correction for ear SRT). The 95% confidence intervals are marked by whiskers. Statistically significant differences at the level of *p* = 0.05 calculated applying Bonferroni correction for multiple comparisons were marked (*).

The 95% confidence intervals for digit-specific and position-specific SRT were determined by bootstrapping and compared applying the Bonferroni correction for multiple comparisons (Figure 5). No statistically significant intra- and inter-group position-specific SRT differences for continuous noise at *p* = 0.05 were found. For modulated noise, only the first position in the control group turned out to be significantly different from zero at the level of 0.36 dB (95% CI 0.18, 0.56) in the absence of inter-group differences at *p* = 0.05. Digit-specific SRT demonstrated intra-group differences that are in line with expectations and are subject to optimization but also inter-group differences. Statistically significant inter-group differences were found for digits 0, 4, 6 and 1, 6 of 1.05 dB, −1.00 dB, −0.91 dB and −1.15 dB, −1,06 dB, respectively, and *p*-values of 0.04, 0.03, 0.05 and 0.05, 0.01 in continuous and modulated noise, respectively (Figure 5, Appendix A).

### 3.2. Adjusted Analysis

Despite statistically significant inter-group digit-specific SRT differences, it can be observed that the trend for each digit is preserved and that the thresholds are similar. Substantial differences in pure-tone audiometry between groups result in slight, but still statistically significant differences in digit-specific SRT. Narrowing the investigated group to subjects whose triplet test results are close to normal may reduce the differences to clinically insignificant values.

**Figure 6:**
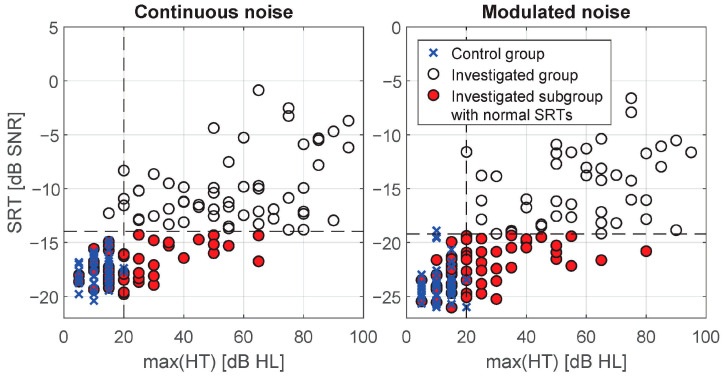
Selection of the investigated subgroup having speech recognition thresholds (SRTs) within normal range. Vertical dashed line—eligibility criteria for the control group; horizontal dashed line—cut-off threshold of normal SRTs; max (HT)—maximal air conduction threshold in the frequency range 0.25–8 kHz.

A subgroup was selected from the investigated group on the basis of test SRT. Ears for which SRT exceeded 99th percentile of the control group were rejected from the investigated group. Finally, 49 out of 112 (43.7%) and 57 out of 112 (50.1%) ears were qualified to the subgroup for continuous and modulated noise, respectively (Figure 6).

The digit-specific SRTs in the control group were compared to the investigated subgroup having SRTs within normal range. The Bonferroni correction for multiple comparisons was applied (Figure 5, Appendix A). For both continuous and modulated noise, no statistically significant inter-groups differences were found at *p* = 0.05.

The decrease in the number of tests in the investigated subgroup resulting from the rejection of tests with SRT above normal may lead to a reduction of the statistical power and consequently to a false negative result. However, the digit-specific deviation of SRT in the full investigated group is greater than in the investigated subgroup, which results in comparable confidence intervals in both groups and ultimately maintains the test power at an unchanged level.

### 3.3. Additional Analysis. The Slope

**Figure 7:**
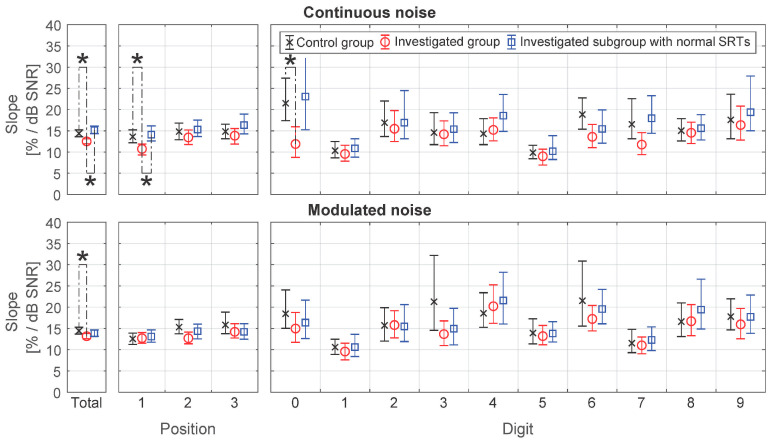
Position-specific and digit-specific slopes of the psychometric function for continuous and modulated noise. The 95% confidence intervals are marked by whiskers. Statistically significant differences at the level of *p* = 0.05 calculated applying Bonferroni correction for multiple comparisons were marked (*).

The digit-specific and position-specific slopes were determined after inter-individual adjustment for SRT analogously to digit-specific and position-specific SRTs. The results are presented in Figure 7 and Appendix B. As expected, in the control group, the slope was significantly higher than in the investigated group for both continuous and modulated noise at *p* < 0.001, d = 1.86 %/dB and *p* = 0.01, d = 1.26 %/dB, respectively. Differences in digit-specific and position-specific slopes were revealed only for the first position (*p* = 0.01, d = 2.9 %/dB) and the digit 0 (*p* = 0.02, d = 9.6 %/dB) in continuous noise, which may be associated with wide confidence intervals that are a consequence of the sample size determined on the basis of the SRT criterion. There were no statistically significant differences at *p* = 0.05 between the control group and the investigated subgroup with normal SRTs for both continuous and modulated noise.

## 4. Discussion

### 4.1. Principal Results

Statistically significant differences in digit-specific SRTs were found at *p* = 0.05 between the control group and the investigated group and were absent when selecting ears having SRT within normal range. This confirms feasibility of digit-specific SRT optimization basing on measurements carried out in a group of subjects with undefined hearing loss, provided ears outside the normal range are rejected.

The position-specific SRT differed significantly from the mean at *p* = 0.05 only in the control group for modulated noise by 0.36 dB (95% CI 0.18–0.56). Given the lack of statistically significant differences for other groups in modulated noise and for all groups in continuous noise, as well as the limited clinical significance of the difference, it can be concluded that the impact of the digit position on the speech intelligibility score is negligible. Therefore position-specific SRTs do not require optimization in this case.

The digit-specific slope differences between the control group and the investigated subgroup with normal SRTs were not found to be statistically significant at *p* = 0.05. Moreover, the mean absolute difference at the level of 1.6%/dB turned out to be much lower than the inter-digit differences up to 10%/dB. Therefore, when optimizing the test, the decision to exclude a digit can be made based on the slope values in the group with normal SRTs.

### 4.2. Comparison with Prior Work

Statistically significant differences in digit-specific SRTs between normal-hearing and hearing-impaired are confirmed by [38]. Wilson and Weakly [38] present SRT levels for English digits in the normal-hearing group and in sensorineural hearing-impaired group with an average hearing threshold in the frequency range 0.25–4 kHz at 40 dB HL. Statistically significant differences at *p* = 0.05 calculated applying the Bonferroni correction for multiple comparisons were obtained for digits 1, 5 and 6. Similar relationships for the same test were found in paper by Wilson et al. [53], in which the largest relative differences were also found for numbers 1 and 5.

The comparison carried out using data from the article by Wilson and Weakly [38] was performed between groups with substantial differences in pure-tone threshold and revealed statistically significant differences in digit-specific SRTs. It should be expected that with lesser differences in the hearing threshold, the differences in digit-specific SRT should also be smaller. In another work by Smits and Houtgast [54], a SRT of the whole triplets was determined in two groups of subjects defined only by means of speech audiometry results. The first group included subjects with SRT from −7.5 to −6.5 dB, which corresponds to the result of normal-hearing subjects, while the second included subjects with SRT from −4.5 to −3.5 dB, which relates to slight hearing loss of average pure-tone threshold in the frequency range 0.5–4 kHz at 25 dB HL [30]. Triplet-specific SRTs in these groups were nearly the same. This remains in line with the present study stating a lack of statistically significant differences between the normal-hearing group verified in pure-tone audiometry and the investigated subgroup having SRTs within normal range. It validates the use of more liberal criteria in qualifying for the group on the basis of which speech material is optimized.

### 4.3. Limitations

This research was carried out only for tests in Polish, which is the limitation of the study. However, considering consistent results for different types of noise and their agreement with English tests [38,53,54], similar relationships should be expected for other languages. Nonetheless, due to the diversity of the world’s languages, the results should be verified each time. In particular, verification of the method is required in all tonal languages. The international recommendations for the development of multilingual speech tests [28,29] do not distinguish between tonal and non-tonal languages. However, the information included in lexical tones significantly influences the mechanisms of speech intelligibility in noise [58,59] and the result of optimization following the recommendations may differ for tonal languages [60]. The rationale for optimization as well as the efficiency of the optimization methods require further research for tonal languages.

No differences in position-specific SRTs were found in this study. The reasons may be due to triplets generated based on non-position-specific recordings of digits, thus missing prosody and coarticulation. The effects of prosody and coarticulation on test efficiency is disputed [29,61] and, along with position optimization, requires further research.

### 4.4. Generalization

Selecting individuals that have SRT within normal range requires prior determination of the test standards in a group on normal-hearing subjects. With limited ability to assess subjects’ hearing thresholds when developing a multilingual test in uncontrolled conditions, it is more efficient to standardize the test and select the measurements within the norms for further optimization as standardization requires fewer measurements than optimization. However, assuming the participation of normal-hearing subjects, it is also possible to perform optimization without prior standardization by using the ears with the lowest SRT in a number not exceeding the number of normal-hearing ears. In other words, when the percentage of normal-hearing subjects in a study population is known, optimization can be performed on up to the same percentage of the best results, as they are bound to be within the norms. The percentage itself, can be easily estimated from, e.g., preliminary measurements or literature data [8] without having to check each subject for a normal hearing threshold.

## 5. Conclusions

The impact of the digit position in the triplet lacking prosody and coarticulation on the speech intelligibility score was found negligible and might not require optimization. Optimization of the digits-specific SRT can be carried out in a group of subjects with undefined hearing loss, provided that the test results outside normal range are rejected. Additionally, if the slope of this digit is much shallower than that of the other digits, excluding the digit can be justified by means of the same group. The findings should be also verified in other languages.

## Figures and Tables

**Table 1:** Baseline demographic data.

Characteristic	Control Group	Investigated Group
Number of participants	26	56
Age (years), range (median)	18–36 (26.5)	21–71(40)
Gender (male), n (%)	12 (48%)	13 (52%)
Number of ears	50	112
Hearing threshold ^1^ (dB HL), mean (SD)	6.3 (6.0)	30.0 (24.7)

^1^ Air conduction at the frequency range 0.25–8 kHz.

**Table 2:** Types of hearing loss in study groups.

Study Group	N	Hearing Loss Type ^1^			
		Normal Hearing	Sensorineural	Conductive	Mixed
Control group	50	50 (100%)	0	0	0
Investigated group	95	34 (36%)	29 (31%)	4 (4%)	28 (29%)
Investigated group with normal SRTs					
Continuous noise	49	30 (61%)	12 (31%)	1 (2%)	7 (12%)
Modulated noise	57	33 (58%)	13 (58%)	2 (4%)	8 (14%)

^1^ Normal hearing: Air conduction (AC) ≤ 20 dB HL for all frequencies, sensorineural hearing loss: AC > 20 dB HL at any frequency and bone gap (BG) < 15 dB HL for all frequencies, conductive hearing loss: BC (bone conduction) ≤ 20 dB HL for all frequencies and BG ≥ 15 dB HL at any frequency, mixed hearing loss: all other ears, AC frequency range 0. 25–8 kHz, BC frequency range 0.25–4 kHz.

## Data Availability

The data presented in this study are available on request from the corresponding author.

## Appendix A

**Table A1:** Position-specific and digit-specific speech reception threshold (SRT).

Characteristic	Control Group	Investigated Group	Investigated Subgroup with Normal Srts
Continuous noise			
N (ears)	50	95	49
SRT (dB SNR), CI (95%), *p*-value ^1^			
Position 1	0.10 (−0.11, 0.30)	0.14 (−0.03, 0.31), 0.83	0.11 (−0.07, 0.28), 0.99
Position 2	−0.02 (−0.20, 0.17)	−0.06 (−0.19, 0.08), 0.78	−0.04 (−0.21, 0.14), 0.90
Position 3	−0.08 (−0.30, 0.15)	−0.16 (−0.30,−0.01), 0.63	−0.01 (−0.19, 0.17), 0.71
Digit 0	0.19 (−0.16, 0.58)	1.24 (0.67, 1.88), 0.04 *	0.74 (0.36, 1.19), 0.17
Digit 1	−0.10 (−0.73, 0.40)	0.49 (−0.00, 1.00), 0.25	0.46 (−0.10, 1.09), 0.31
Digit 2	−1.40 (−1.80,−0.99)	−1.17 (−1.55,−0.80), 0.57	−1.10 (−1.64, −0.72), 0.55
Digit 3	−0.27 (−0.81, 0.23)	−0.53 (−0.89,−0.17), 0.59	−0.32 (−0.75, 0.14), 0.92
Digit 4	1.11 (0.69, 1.63)	0.11 (−0.24, 0.57), 0.03 *	0.45 (0.01, 0.91), 0.15
Digit 5	0.37 (−0.32, 1.13)	1.06 (0.54, 1.56), 0.31	0.26 (−0.30, 1.01), 0.88
Digit 6	−0.03 (−0.45, 0.41)	−0.94 (−1.41, −0.48), 0.05 *	−0.15 (−0.66, 0.42), 0.82
Digit 7	0.20 (−0.16, 0.57)	0.75 (0.32, 1.25), 0.18	0.52 (0.14, 0.92), 0.41
Digit 8	0.23 (−0.18, 0.64)	0.04 (−0.30, 0.41), 0.64	0.21 (−0.28, 0.67), 0.96
Digit 9	−0.16 (−0.54, 0.15)	−0.74 (−1.13, −0.40), 0.12	−0.58 (−1.04, −0.19), 0.29
Total ^2^	0.00 (−0.09, 0.09)	−0.03 (−0.11, 0.06), 0.65	0.02 (−0.07, 0.11), 0.79
Modulated noise			
N (ears)	50	90	57
SRT (dB SNR), CI (95%), *p*-value ^1^			
Position 1	0.36 (0.18, 0.56)	0.20 (0.02, 0.35), 0.26	0.21 (0.01, 0.40), 0.36
Position 2	−0.16 (−0.35, 0.04)	−0.12 (−0.26, 0.03), 0.80	−0.07 (−0.25, 0.13), 0.60
Position 3	−0.10 (−0.27, 0.10)	−0.09 (−0.23, 0.06), 0.94	−0.13 (−0.30, 0.05), 0.83
Digit 0	1.37 (0.95, 1.86)	1.55 (1.16, 2.10), 0.70	1.53 (1.05, 2.00), 0.75
Digit 1	2.87 (2.34, 3.56)	1.72 (1.19, 2.31), 0.05 *	2.01 (1.36, 2.64), 0.15
Digit 2	−1.36 (−1.86, −0.87)	−1.80 (−2.19, −1.44), 0.33	−1.61 (−2.06, −1.18), 0.61
Digit 3	−1.61 (−2.07, −1.21)	−0.84 (−1.25, −0.31), 0.07	−1.37 (−1.78, −0.77), 0.57
Digit 4	0.02 (−0.33, 0.37)	0.13 (−0.15, 0.44), 0.74	0.02 (−0.34, 0.44), 0.99
Digit 5	0.54 (−0.03, 1.04)	0.69 (0.26, 1.05), 0.75	0.48 ( 0.02, 0.96), 0.91
Digit 6	0.10 (−0.28, 0.40)	−0.96 (−1.30, −0.58), 0.01 *	−0.67 (−1.08, −0.25), 0.06
Digit 7	−1.12 (−1.69, −0.60)	−0.18 (−0.65, 0.33), 0.06	−0.32 (−0.81, 0.26), 0.12
Digit 8	0.32 (−0.04, 0.72)	0.11 (−0.20, 0.41), 0.53	0.41 ( 0.07, 0.76), 0.82
Digit 9	−0.15 (−0.48, 0.13)	−0.13 (−0.43, 0.24), 0.95	−0.19 (−0.56, 0.18), 0.82
Total ^2^	0.02 (−0.07, 0.11)	−0.00 (−0.08, 0.07), 0.68	0.00 (−0.09, 0.09), 0.75

^1^*p*-value for comparison with the control group. Statistically significant differences at *p* = 0.05 after applying the Bonferroni correction to account for simultaneous comparison of 3 positions and simultaneous comparison of 10 digits were marked by (*). ^2^ SRT calculated using psychometric function fitted to all data points may differ from the average of single measurements, therefore the values are not exactly 0.

## Appendix B

**Table A2:** Position-specific and digit-specific slopes of the psychometric function.

Characteristic	Control Group	Investigated Group	Investigated Subgroup with Normal Srts
Continuous noise			
N (ears)	50	95	49
Slope (%/dB), CI (95%), *p*-value ^1^			
Position 1	13.61 (12.19, 15.24)	10.73 (9.32, 11.88), 0.01 *	14.09 (12.61, 16.17), 0.73
Position 2	14.82 (12.89, 16.83)	13.44 (11.75, 15.17), 0.39	15.30 (13.63, 17.53), 0.77
Position 3	14.83 (13.14, 16.56)	13.85 (11.89, 15.54), 0.51	16.34 (14.28, 18.94), 0.37
Digit 0	21.50 (17.40, 27.40)	11.90 (8.73, 15.69), 0.02 *	23.08 (15.27, 32.17), 0.83
Digit 1	10.33 (8.64, 12.48)	9.55 (7.84, 11.59), 0.69	10.87 (8.78, 13.13), 0.81
Digit 2	16.90 (13.67, 22.01)	15.48 (12.49, 19.78), 0.72	16.92 (13.13, 24.49), 0.99
Digit 3	14.60 (11.74, 19.28)	14.19 (11.47, 17.35), 0.89	15.40 (12.24, 19.24), 0.85
Digit 4	14.33 (11.74, 17.87)	15.22 (12.66, 18.04), 0.78	18.56 (14.85, 23.56), 0.26
Digit 5	9.87 (8.41, 11.60)	9.00 (6.92, 10.69), 0.59	10.19 (8.24, 13.86), 0.87
Digit 6	18.84 (15.39, 22.76)	13.59 (11.04, 16.53), 0.11	15.45 (12.08, 19.95), 0.41
Digit 7	16.54 (13.12, 22.60)	11.76 (9.40, 14.58), 0.14	17.96 (14.41, 23.27), 0.78
Digit 8	15.04 (12.59, 17.87)	14.54 (12.01, 17.04), 0.84	15.62 (12.84, 18.82), 0.84
Digit 9	17.57 (13.15, 23.63)	16.37 (12.80, 20.81), 0.79	19.38 (15.03, 27.9), 0.74
Total	14.36 (13.59, 15.23)	12.50 (11.96, 13.09), <0.001 *	15.17 ^2^ (14.33, 16.11), 0.19
Modulated noise			
N (ears)	50	90	57
Slope (%/dB), CI (95%), *p*-value ^1^			
Position 1	12.55 (11.27, 13.91)	12.73 (11.53, 14.06), 0.88	13.11 (11.78, 14.66), 0.64
Position 2	15.33 (13.75, 17.10)	12.71 (11.41, 14.16), 0.05	14.37 (12.56, 16.03), 0.50
Position 3	15.85 (13.78, 18.82)	14.22 (12.77, 15.15), 0.36	14.21 (12.47, 16.12), 0.35
Digit 0	18.44 (15.05, 24.06)	14.99 (11.76, 18.76), 0.35	16.36 (12.62, 21.62), 0.65
Digit 1	10.61 (8.90, 12.47)	9.58 (7.62, 11.54), 0.59	10.62 (8.41, 13.64), 0.99
Digit 2	15.72 (12.02, 19.89)	15.80 (12.81, 19.15), 0.98	15.48 (11.93, 20.62), 0.96
Digit 3	21.27 (14.57, 32.20)	13.68 (10.99, 16.76), 0.16	14.97 (11.13, 19.72), 0.29
Digit 4	18.54 (15.28, 23.40)	20.21 (16.22, 25.25), 0.72	21.56 (16.07, 28.19), 0.57
Digit 5	13.93 (11.37, 17.26)	13.20 (11.16, 15.72), 0.78	13.81 (11.82, 16.57), 0.97
Digit 6	21.50 (15.57, 30.85)	17.24 (14.45, 20.38), 0.38	19.55 (16.12, 24.19), 0.72
Digit 7	11.55 (9.31, 14.79)	11.03 (9.04, 13.00), 0.81	12.31 (9.81, 15.36), 0.80
Digit 8	16.60 (13.09, 21.01)	16.70 (13.31, 20.59), 0.98	19.41 (14.86, 26.60), 0.54
Digit 9	17.76 (14.69, 21.95)	15.95 (12.58, 19.68), 0.61	17.70 (13.87, 22.84), 0.99
Total	14.44 (13.66, 15.31)	13.18(12.61, 13.81), 0.01 *	13.87 (13.16, 14.66), 0.31

^1^*p*-value for comparison with the control group. Statistically significant differences at *p* = 0.05 after applying the Bonferroni correction to account for simultaneous comparison of 3 positions and simultaneous comparison of 10 digits were marked by (*). ^2^ The slope in the investigated subgroup with normal SRTs may be even greater compared to the control group because steep slopes are observed mostly at low SRT.
